# Supplementary material for: The State of the Dopaminergic and Glutamatergic Systems in the Valproic Acid Mouse Model of Autism Spectrum Disorder
Source: Biomolecules. 2022 Nov 15;12(11):1691. doi: 10.3390/biom12111691 (PMC9688008; doi:10.3390/biom12111691)
Supplement: Supplementary file 1 [file biomolecules-12-01691-s001.zip › biomolecules-2004666-supplementary.pdf]

Table S1: Primers sequences used for PCRq experiments.

Targets	Forward primers	Reverse primers
**GAPDH**	gtcggtgtgaacggattt	ccatttgatgttagtggggt
**NR1**	catcggacttcagctaatca	gtccccatcctcattgaatt
**NR2A**	ggctacagagacttcatcag	atccagaagaaatcgtagcc
**NR2B**	ccatcattctcctctactgc	caatcccatctctcactctg
**mGluR1**	tgaagtcatcgaaggctatg	ccaagctttcatttcctgtg
**mGluR4**	gtcaacttctcaggcatcg	tctattctgaggtgcaagtg
**mGluR5**	aggacagataaaggtgatcc	agatactggactgggatcaa
**DRD1**	ctctgccctacaacgaataa	catcggtgtcatagtccaat
**DRD2**	ctttgtcactctggatgtca	cattctggtctgtgttgttg
**DRD3**	gacaaaccatatccccagaa	tttcgaacctctaagctgag
